# Understanding voltage-controlled magnetic anisotropy effect at Co/oxide interface

**DOI:** 10.1038/s41598-023-37422-4

**Published:** 2023-06-30

**Authors:** Tomohiro Nozaki, Jun Okabayashi, Shingo Tamaru, Makoto Konoto, Takayuki Nozaki, Shinji Yuasa

**Affiliations:** 1 Research Center for Emerging Computing Technologies (RCECT), National Institute of Advanced Industrial Science and Technology (AIST), Tsukuba, Ibaraki 305-8568 Japan; 2 Research Center for Spectrochemistry, The University of Tokyo, Tokyo, 113-0033 Japan

**Keywords:** Electronic devices, Electrical and electronic engineering, Magnetic properties and materials

## Abstract

The voltage-controlled magnetic anisotropy (VCMA) effect is a key to realising high-speed, ultralow-power consumption spintronic devices. The fcc-Co-(111)-based stack is a promising candidate for the achievement of large VCMA coefficients. However, only a few studies on the fcc-Co-(111)-based stack have been reported and the VCMA effect has not been well understood. Previously, we observed a significant increase in the voltage-controlled coercivity (VCC) in the Pt/Ru/Co/CoO/TiO_*x*_ structure upon post-annealing. However, the mechanism underlying this enhancement remains unclear. This study performs multiprobe analyses on this structure before and after post-annealing and discusses the origin of the VCMA effect at the Co/oxide interface. X-ray magnetic circular dichroism measurement revealed an increase in the orbital magnetic moment owing to post-annealing, accompanied by a significant increase in VCC. We speculate that the diffusion of Pt atoms into the vicinity of Co/oxide interface enhances the interfacial orbital magnetic moment and the VCMA at the interface. These results provide a guideline for designing structures to obtain a large VCMA effect in fcc-Co-(111)-based stacks.

## Introduction

Electric-field control of magnetism has attracted significant attention because it can realize ultralow-power spintronics devices. The voltage-controlled magnetic anisotropy (VCMA) effect^1–5^ is a high-speed spin manipulation technique with excellent compatibility with magnetoresistive random access memory (MRAM). It has been studied for applications such as voltage-controlled MRAM^4,6^. VCMA effect has been mainly investigated at transition metal (TM) ferromagnet/oxide dielectric interfaces, such as bcc-Fe (001)/MgO and fcc-Co (111)/oxide interfaces, where the hybridisation of TM 3*d* and O 2*p* orbitals produces a strong interface perpendicular magnetic anisotropy (PMA)^7–10^*.* In particular, bcc-Fe-(001)-based stacks have been investigated extensively and a VCMA coefficient of 350 fJ/Vm has been achieved using high-quality epitaxial films^11–13^. However, further improvement in the VCMA coefficient is demanded. The VCMA effect was also investigated for fcc-Co-(111)-based stacks. In these stacks, a large VCMA coefficient of 230 fJ/Vm was reported, even for polycrystalline film^14^, indicating that the VCMA coefficient can be further improved. However, in fcc-Co-based stacks, systematic studies of the VCMA effect have not been conducted and the understanding is still in its infancy compared with that of bcc-Fe-based stacks. In fcc-Co-based stacks, a particularly large VCMA effect, including voltage-controlled coercivity (*H*_c_) (VCC), has been reported at interfaces with surface oxidation of Co^14–18^, suggesting the significant role of surface oxidation. In our previous study^18^, we observed a large VCC in the Pt/Co/CoO/TiO_*x*_ structure. Furthermore, we investigated a VCC in the Pt/Co/Ru/CoO/TiO_*x*_ structure, where the Ru-inserted layer decreased the interface PMA at the Pt/Co lower interface. The VCC was significantly enhanced by optimal post-annealing. However, the reasons for the improvement in VCC by post-annealing remain unclear.

Although few studies on the VCMA effect have been conducted, Pt/Co/AlO_*x*_ trilayers have long been focused on the studies of large interface PMA, strong Rashba effect, and Dzyaloshinskii–Moriya interaction^19–25^. The effect of the oxidation conditions on the interface PMA was examined in detail using Pt/Co/post-oxidised Al trilayers^19^. The PMA at the Co/oxide interface was maximised under appropriate oxidation conditions^26–29^ with the Co–O bonds at the interface^28^ and enhanced by post-annealing^29^. In addition, an increase in the orbital magnetic moment (*m*_orb_) at the Co/oxide interface was observed^30,31^. Based on Bruno’s theory, the anisotropy of *m*_orb_ corresponds to the stabilisation of PMA^32^. Interface PMA in 3*d* TM ferromagnets is often discussed based on Bruno’s theory^3,10,33–35^. Thus, the increase in the interface PMA and interfacial *m*_orb_ observed in Pt/Co/AlO_*x*_ trilayers may also be linked to the large VCMA effect obtained at surface-oxidised Co/oxide interfaces. However, the VCMA effect on fcc-Co-based stacks has not been discussed in relation to previous studies on interface PMA in Pt/Co/AlO_*x*_ trilayers. In addition, studies of post-annealing effects on the interface PMA or VCMA effect in Pt/Co/oxide trilayers are limited^18,29^, whereas post-annealing is an effective method of improving the quality of the interface and enhancing interfacial phenomena.

In this study, to understand VCMA effect at fcc-Co (111)/oxide interfaces, we systematically investigated the dependence of the VCC in the Pt/Ru/Co/CoO/TiO_*x*_ structure on Co film thickness and annealing temperature. The Ru-inserted layer decreased the PMA (or *H*_c_) and VCC in the as-deposited state; however, post-annealing significantly enhanced the VCC in this structure. Therefore, we qualitatively discussed the effect of post-annealing on VCC by examining the systematic changes in the structure, capacitance, and magnetic state before and after post-annealing. We proposed a structure with a large VCMA effect in fcc-Co-(111)-based stacks based on these results.

## Results

**Figure 1:**
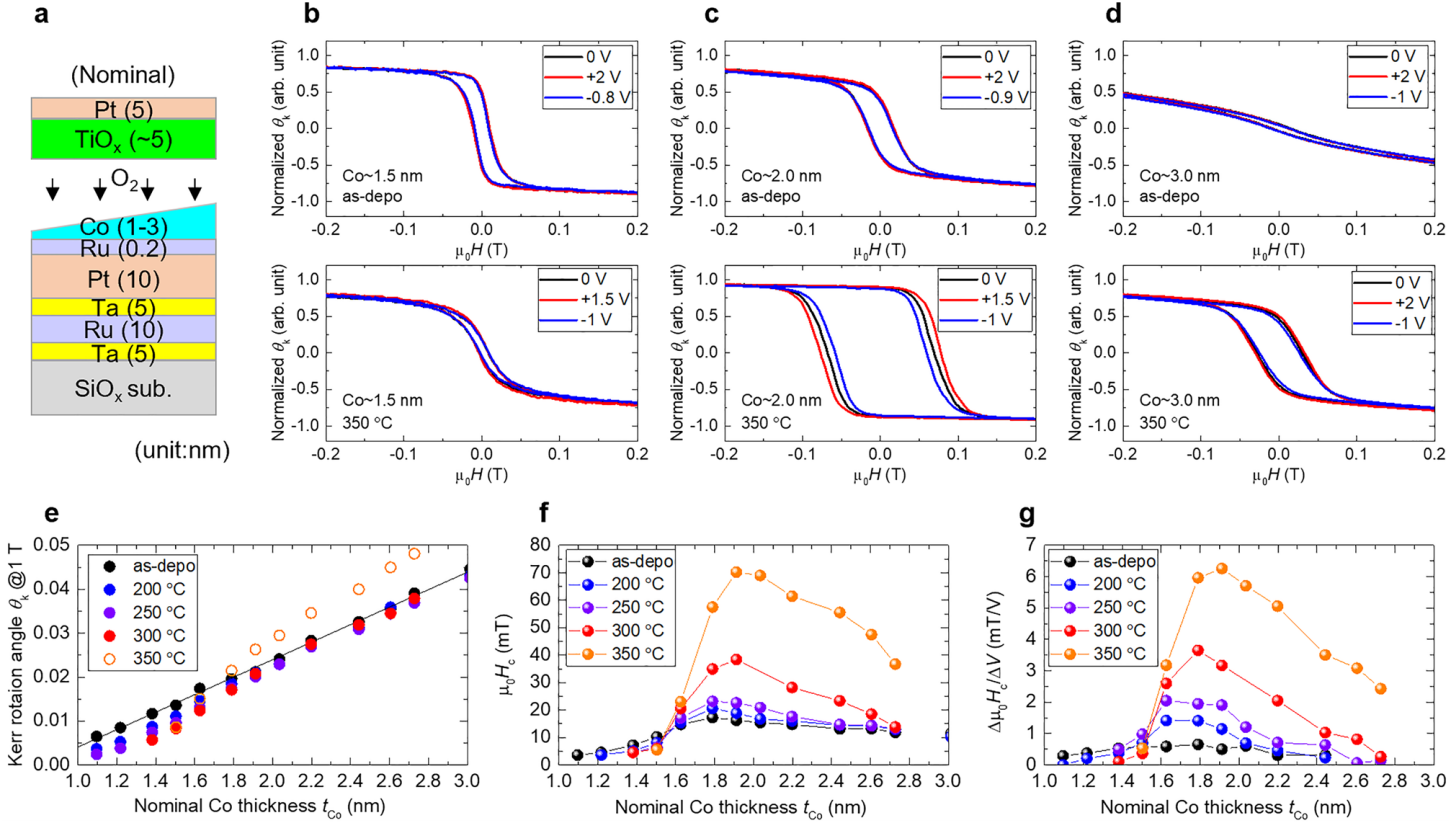
(**a**) Schematics of the nominal structure of the sample for MOKE measurements. (**b**–**d**) Normalised perpendicular magnetisation curves under bias-voltages measured by MOKE, for the sample with *t*_Co_ = (**b**) 1.5 nm, (**c**) 2.0 nm, and (**d**) 3.0 nm. The upper (lower) panel shows the results of the as-deposited (350 °C-annealed) samples. The black, red, and blue lines correspond to the magnetisation curve under zero, positive, and negative bias-voltages, respectively. (**e**–**g**) *t*_Co_ dependence of (**e**) *θ*_k_, (**f**) *H*_c_, and (**g**) Δ*H*_c_/Δ*V* for various annealing temperatures.

We measured the bias-voltage dependence of the perpendicular magnetisation curve of the nominal SiO_*x*_ sub./Ta (5 nm)/Ru (10 nm)/Ta (5 nm)/Pt (10 nm)/Ru (0.2 nm)/Co wedge (*t*_Co_ nm)/TiO_*x*_ (approximately 5 nm)/Pt (5 nm) structure (Fig. 1a) using magneto-optical Kerr effect (MOKE). In this structure, part of the Co was oxidised to CoO during TiO_*x*_ deposition. From the thickness dependence of the magnetisation curve of similar samples measured using a vibrating sample magnetometer (VSM), approximately 1.0 nm of Co loses magnetisation owing to oxidation (Supplementary Information S1). The actual structure was assumed to be Pt/Ru/Co (*t*_Co_* = *t*_Co_ − 1.0 nm)/CoO (1.8 nm)/TiO_*x*_. Here, *t*_Co_ and *t*_Co_* represent the nominal Co thickness and the assumed unoxidised Co thickness, respectively. The *t*_Co_ varied from 1.0 to 3.0 nm and the unoxidised Co thickness *t*_Co_* ranged from 0 to 2.0 nm. The perpendicular magnetisation curves under bias-voltage for samples with *t*_Co_ = 1.5 nm, 2.0 nm, and 3.0 nm are shown in Fig. 1b–d. The results of the as-deposited samples are shown in the upper panel and the results of the 350 °C-annealed samples are shown in the lower panel. The black, red, and blue lines correspond to magnetisation curves under zero, positive, and negative bias-voltages, respectively. The magnetisation curve in Fig. 1b–d is normalised because the Kerr rotation angle (*θ*_k_) may not correspond to the magnitude of magnetisation, as discussed in Supplementary Information S1 and S2. For the as-deposited samples, a magnetisation curve with relatively good squareness was observed in the thin Co region (*t*_Co_ = 1.5 nm). The squareness decreased with increasing Co thickness and was completely lost in the thick Co region (*t*_Co_ = 3.0 nm). This tendency indicated the dominant contribution of the interface PMA to the magnetic anisotropy of the sample. The interface PMA arise from both the upper and lower interface of Co. Since the interface PMA at the Pt/Co lower interface was weakened by Ru insertion, we assumed that the interface PMA at the Co/oxide upper interface was predominant. For as-deposited samples, a slight change in *H*_c_ (*t*_Co_ = 1.5 and 2.0 nm) or anisotropy field *H*_k_ and squareness (*t*_Co_ = 2.0 and 3.0 nm) were observed with the application of a bias-voltage. For the 350 °C-annealed samples, compared with as-deposited samples, significant improvements in the *H*_c_ and squareness were observed in the thicker Co region (*t*_Co_ = 2.0 and 3.0 nm), suggesting the interface PMA was enhanced by post-annealing. In the sample with *t*_Co_ = 2.0 nm, where a good squareness and large *H*_c_ were obtained, a large VCC of − 5.7 mT/V was obtained. However, in the thin Co region (*t*_Co_ = 1.5 nm), the squareness, *H*_c_, and VCC were degraded compared with the as-deposited sample. The *t*_Co_ dependence of *θ*_k_ @1 T at various annealing temperatures is shown in Fig. 1e. As discussed in Supplementary Information S2, the magnitude of *θ*_k_ approximately corresponds to the magnitude of magnetisation, except for the 350 °C-annealed samples. In the case of as-deposited samples, *θ*_k_ increased linearly with *t*_Co_. The fitted line is indicated by the black line in Fig. 1e. After post-annealing, in the thick Co region, *θ*_k_ slightly decreased uniformly. In contrast, in the thin Co region, *θ*_k_ decreased significantly. The region in which a significant decrease in *θ*_k_ occurred expanded with an increase in the annealing temperature. The *t*_Co_ dependence of *H*_c_ and VCC (Δ*H*_c_/Δ*V* = *H*_c_@1 V − *H*_c_@0 V) for various annealing temperatures is shown in Fig. 1f,g. The PMA of the as-deposited sample was small owing to Ru insertion and only a small *H*_c_ was obtained regardless of the Co thickness. Post-annealing significantly improved the PMA in the thick Co region and *H*_c_ was enhanced with an increasing annealing temperature. A similar but more significant enhancement was observed for the VCC. For example, in the sample with *t*_Co_ = 1.9 nm, the *H*_c_ and VCC were enhanced by a factor of 4.3 (16.3 mT to 70.3 mT) and 12.5 (0.50 mT/V to 6.26 mT/V), respectively. However, *H*_c_ and VCC degraded in the thin Co region (1.5 nm ≤ *t*_Co_) owing to post-annealing. The Co region, in which *H*_c_ and VCC degraded (Fig. 1f,g), coincided with the region in which *θ*_k_ decreased (Fig. 1e). Note that VCC was underestimated in the thick Co region in which the Co magnetisation curve exhibited poor squareness or had in-plane magnetisation components. In such a Co region, both *H*_c_ and *H*_k_ change with voltage application. In addition, *H*_c_ decreased in the thicker Co region because the interface PMA was dominant. Therefore, *H*_c_ and VCC decreased in the thick Co region. Note that *H*_c_ and VCC do not have a quantitative one-to-one correspondence with PMA and VCMA. In the past, even a difference in the sign between VCC and VCMA has been reported^36^. In this study we observed a post-annealing induced enhancement of the *H*_c_ accompanied by an improvement of the squareness in *e.g.* Figure 1c,d confirming the qualitative correspondence of *H*_c_ and PMA. We also observed simultaneous enhancement of the *H*_c_ and improvement of the squareness by applying a positive bias voltage in e.g. Figure 1d, confirming the coincidence of the sign between VCC and VCMA. We’ve also confirmed the linear variation of *H*_c_ against bias-voltage^18^. Thus the post-annealing induced enhancement of VCC may primarily reflects the enhancement of VCMA.

**Figure 2:**
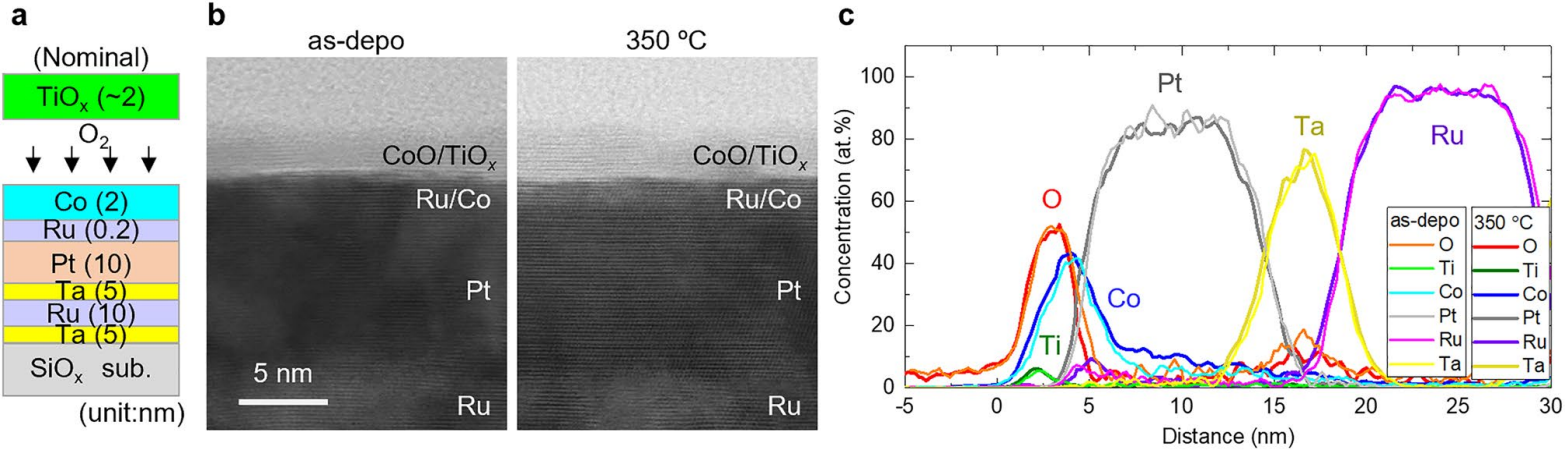
(**a**) Schematics of nominal structures of the samples for STEM measurements. (**b**) STEM images and (**c**) EDX line profile of the as-deposited and 350 °C-annealed samples.

Cross-sectional scanning transmission electron microscopy (STEM) analysis was performed on the as-deposited and 350 °C-annealed samples with *t*_Co_ = 2.0 nm to investigate the post-annealing-induced structural change. The nominal sample structure is shown in Fig. 2a. The sample used for the STEM analysis is identical to the sample used for the X-ray absorption spectroscopy (XAS)/X-ray magnetic circular dichroism (XMCD) measurements and had a thinner TiO_*x*_ thickness compared with the sample used for the MOKE measurement. The bright-field STEM images before and after post-annealing are shown in Fig. 2b. Identifying the thicknesses of the Co and dielectric layers from the STEM images was difficult because of their thin thickness and similar contrast to the adjacent layers. The thicknesses of the other layers were the same as the nominal thickness. No noticeable changes in the film structure were observed before and after the post-annealing. The energy dispersive X-ray spectroscopy (EDX) line profiles of the sample before and after the post-annealing are shown in Fig. 2c. The corresponding EDX element map are shown in Supplementary Information S3. In Fig. 2c, the distance of 0 nm corresponds to the upper edge of the TiO_*x*_ layer. The EDX line profile provides depth-averaged information, including the influence of roughness at the interfaces. Taking this into account, the line profiles were almost identical before and after the post-annealing. However, a slight but obvious difference was observed in the Co spectrum; the Co spectrum after annealing (blue line) spread toward the Pt layer compared with that before annealing (light blue line), indicating the diffusion of Co atoms to the Pt layer. The diffusion of Pt atoms into the Co layer was not clearly visible because of the thin Co thickness and roughness of the polycrystalline sample. However, because Co and Pt are rather miscible^37^, interdiffusion of Co and Pt was anticipated. The effect of the Ru-inserted layer on the interdiffusion of Co and Pt in the Pt/Ru/Co/TiO_*x*_ structure is discussed in Supplementary Information S5. The diffusion of Pt atoms into the Co layer could be validated from STEM analysis of epitaxial films, which is considered in future studies. The off-trend change in *θ*_k_ after annealing at 350 °C (Fig. 1e) possibly resulted from the mixing of Co and Pt. Despite the possible interdiffusion of Co and Pt, the Ru between them does not mix with Co and Pt after annealing at 350 °C (purple line) and existed in almost the same position as before annealing (or move slightly toward the Pt layer) (pink line). The post-annealing effect on the dielectric layers (CoO and TiO_*x*_) was not obviously captured because of the thin dielectric layer thickness.

**Figure 3:**
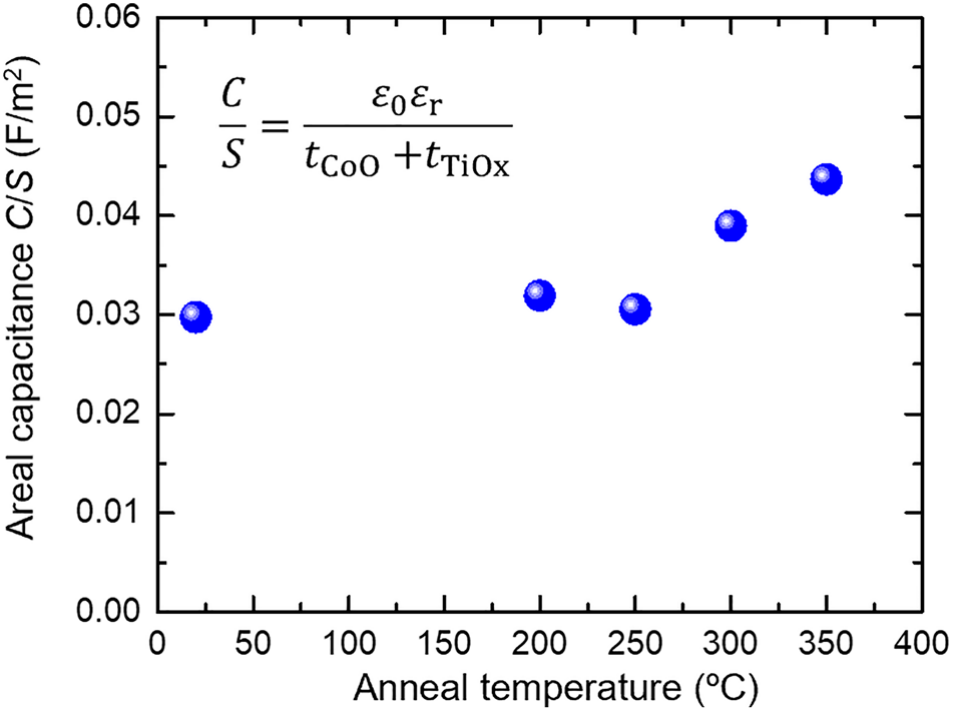
Annealing temperature dependence of the *C*/*S* of the nominal SiO_*x*_ sub./Ta (5 nm)/Ru (10 nm)/Ta (5 nm)/Pt (10 nm)/Ru (0.2 nm)/Co (3 nm)/TiO_*x*_ (approximately 5 nm)/Pt (5 nm) structure.

**Figure 4:**
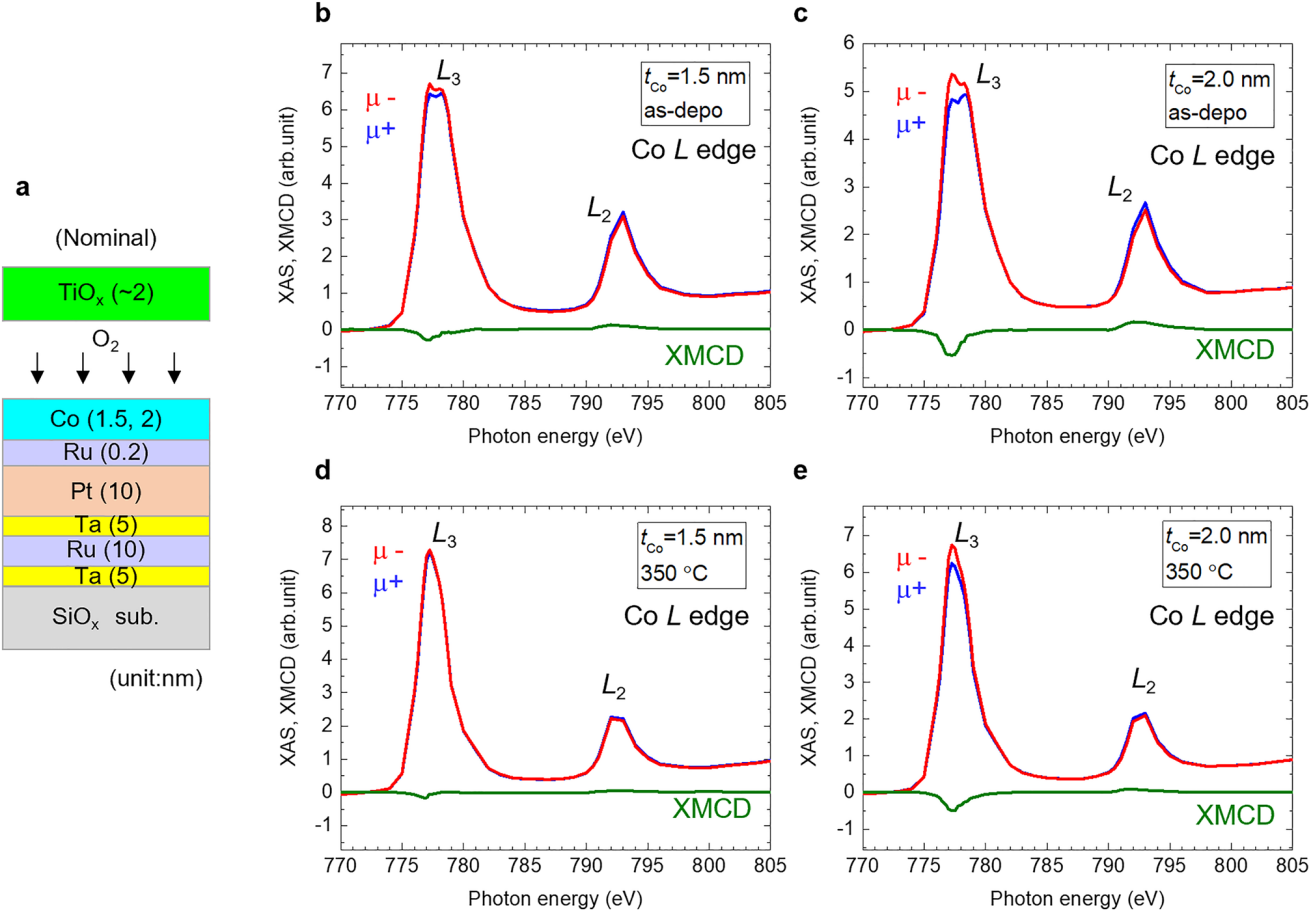
(**a**) Schematics of nominal structures of samples for XAS/XMCD measurements. (**b**)–(**e**) Normalised Co *L*-edge XAS and XMCD spectra of as-deposited samples for *t*_Co_ = (**b**) 1.5 nm and (**c**) 2.0 nm, and 350 °C-annealed samples for *t*_Co_ = (**d**) 1.5 nm, and (**e**) 2.0 nm. The red and blue lines are XAS spectra depending on the magnetic field by fixed helicity (*μ−* and *μ*+). The green lines represent the XMCD spectra.

Capacitance measurements were performed on the microfabricated samples to investigate the changes in the CoO/TiO_*x*_ dielectric layers owing to post-annealing. The annealing temperature dependence of the areal capacitance *C*/*S* of the nominal SiO_*x*_ sub./Ta (5 nm)/Ru (10 nm)/Ta (5 nm)/Pt (10 nm)/Ru (0.2 nm)/Co (3 nm)/TiO_*x*_ (approximately 5 nm)/Pt (5 nm) is shown in Fig. 3. Parasitic capacitance components^38^ were subtracted by measuring the element size dependence of *C*/*S*. *C*/*S* increased at annealing temperatures above 300 °C and reached to value approximately 1.5 times higher compared with that of the as-deposited sample after annealing at 350 °C. Because the areal capacitance is represented as *C*/*S* = *ε*_0_*ε*_r_/(*t*_CoO_ + *t*_TiOx_), the increase of *C*/*S* may reflect a decrease in the total dielectric layer thickness (*t*_CoO_ + *t*_TiOx_) and an improvement in the dielectric constant *ε*_r_ by post-annealing. Although not clearly visible from the STEM-EDX analysis, as discussed later, XAS spectra in Fig. 4 suggest a partial reduction in the CoO layer by post-annealing, resulting in a decrease in the dielectric layer thickness. In addition, the overall *ε*_r_ (estimated to be *ε*_r_ ~ 29 in our previous study^18^) can be improved, *e.g.* through the formation of a high *ε*_r_ CoTiO_3_ (*ε*_r_ ~ 45)^39^ or a decrease in the low *ε*_r_ CoO thickness (*ε*_r_ ~ 13)^40^. In general, an increase in *C*/*S*, *i.e.* increase in accumulated charge per unit voltage, leads to an increase in VCC. However, in this study, the contribution of the post-annealing induced increase in *C/S* to the increase in VCC (Fig. 1g) is small; the increment in *C*/*S* (1.5 times) is much smaller than the increment in VCC (up to 12.5 times).

XAS/XMCD measurements were performed for samples of the nominal SiO_*x*_ sub./Ta (5 nm)/Ru (10 nm)/Ta (5 nm)/Pt (10 nm)/Ru (0.2 nm)/Co (*t*_Co_ nm)/TiO_*x*_ (approximately 2 nm) structures (*t*_Co_ = 1.5 and 2.0) to investigate the element-specific chemical and magnetic states in the Co sites. The sample structures are shown in Fig. 4a. The measurement geometries were set to normal incidence, such that the directions of the photon helicity axis and magnetic field were parallel and normal to the surface, enabling the measurement of the absorption processes involving the normal components of the spin (*m*_spin_) and orbital magnetic moments (*m*_orb_). The Co *L*-edge XAS and XMCD spectra of as-deposited samples are shown in Fig. 4b,c. All spectra were normalised to the post-edge. The XAS signals comprised metallic Co (777.0 eV) and chemically shifted CoO (Co^2+^) components (778.0 eV). Metallic Co components of the XAS and XMCD signals increased with an increasing *t*_Co_. The magnitude of the XMCD signal almost corresponded to that of magnetisation (Fig. S1b in Supplementary Information S1). The Co *L*-edge XAS and XMCD spectra of the 350 °C-annealed samples are shown in Fig. 4d,e. A clear change in the XAS spectral line shapes was observed; the CoO component at 778.0 eV decreased owing to annealing at 350 °C. Note that while the spectra after annealing appeared as a single peak of metallic Co component, the spectra contain the CoO component; the peak at 777.0 eV broadened compared with that of metallic Co (Supplementary Information S4), suggesting the partial reduction of the CoO layer owing to post-annealing. A similar reduction in CoO by post-annealing, accompanied by Pt atoms diffusion, was previously discussed^29^. Despite the reduction in CoO, no significant increase in XMCD signals was observed; the sample with *t*_Co_ = 2.0 nm showed an XMCD intensity comparable to that of the as-deposited sample. The sample with *t*_Co_ = 1.5 nm showed an XMCD intensity smaller than that of the as-deposited sample. These can be attributed to the decrease in Co owing to the diffusion of Co atoms into the Pt layer, as revealed by STEM-EDX analysis.

**Figure 5:**
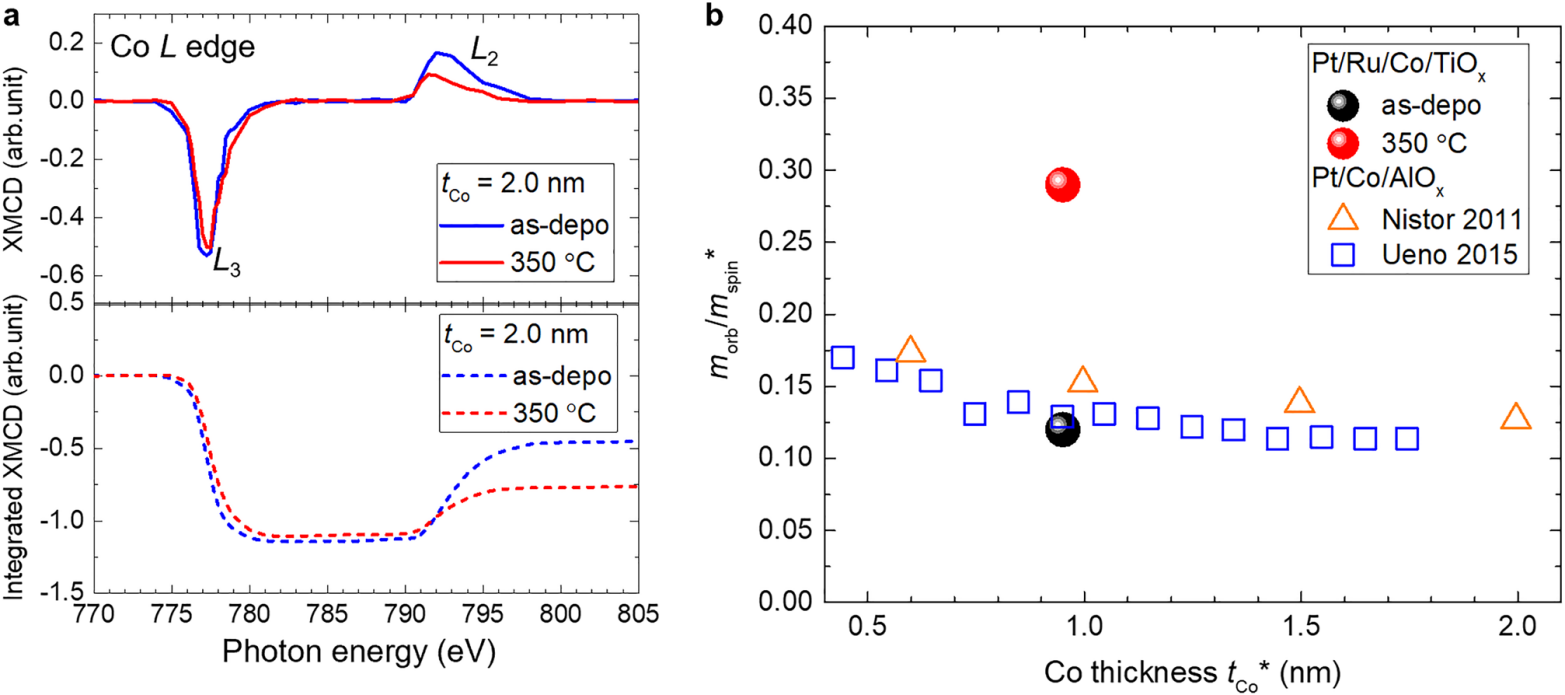
(**a**) Normalised Co *L*-edge XMCD spectra and integrated XMCD spectra of as-deposited and 350 °C-annealed samples of *t*_Co_ = 2.0 nm. (**b**) Co thickness dependence of *m*_orb_/*m*_spin_* obtained in this study (Pt/Ru/Co/TiO_*x*_ structure) and previous studies (Pt/Co/AlO_*x*_ structure)^30,31^.

Next, the magnetic moments were examined using the magneto-optical sum rule analysis. *m*_orb_ and effective spin magnetic moment (*m*_spin_*) cannot be evaluated explicitly from the integrals of the XAS and XMCD spectra because the Co *L*-edge XAS spectra included Co and CoO components. However, the ratio of *m*_orb_/*m*_spin_* can be estimated from the XMCD line shape integrals using the following equation, assuming XMCD spectra originate from metallic Co without oxidation^41^:

1 $$\frac{{m_{orb} }}{{m_{spin} *}} = \frac{2}{3}\frac{{A_{L3} + A_{L2} }}{{A_{L3} - 2A_{L2} }}$$

where *A*_L3_ and *A*_L2_ are the integrated areas of the *L*_3_ and *L*_2_ peaks in the XMCD spectrum, respectively. Assuming CoO components do not contribute to XMCD signals, we evaluated *m*_orb_/*m*_spin_* for as-deposited and 350 °C-annealed samples with *t*_Co_ = 2.0 nm, for which sufficient XMCD intensity was obtained. The Co *L*-edge XMCD and the integrated spectra of the as-deposited and 350 °C-annealed samples with *t*_Co_ = 2.0 nm are shown in Fig. 5a. A clear difference in *L*_2_ peak intensity before and after annealing was observed. Based on Eq. (1), the ratio *m*_orb_/*m*_spin_* increased for small *L*_2_ peak intensity or large residuals of the integrals of *L*_3_ and *L*_2_ edges in XMCD spectra. *m*_orb_/*m*_spin_* values evaluated using Eq. (1) were 0.12 and 0.29 for the as-deposited and 350 °C-annealed samples with *t*_Co_ = 2.0 nm, respectively. The post-annealing induced increase in *m*_orb_/*m*_spin_* corresponds to the increase in the *H*_c_ and VCC and may occurs at the Co/oxide upper interface, as discussed later. The Co thickness dependence of *m*_orb_/*m*_spin_* obtained in this study (Pt/Ru/Co/TiO_*x*_ structure) and previous studies (Pt/Co/AlO_*x*_ structure) is summarized in Fig. 5b^30,31^. In the previous Pt/Co/AlO_*x*_ trilayer structure, an increase in *m*_orb_/*m*_spin_* from a bulk value^41^ of approximately 0.10 with decreasing Co thickness was estimated, indicating the existence of an interfacial *m*_orb_ in this structure^30,31^. Because such an increase in *m*_orb_/*m*_spin_* was not observed in the Pt/Co/Pt trilayer structure^31^, the interfacial *m*_orb_ may arise from the Co/oxide upper interface, rather than at the Pt/Co lower interface. In this study, we observed *m*_orb_/*m*_spin_* comparable to those reported in the Pt/Co/AlO_*x*_ structure^30,31^ for as-deposited Pt/Ru/Co/TiO_*x*_ structures. In addition, we validated an increase in *m*_orb_/*m*_spin_* in the Pt/Ru/Co/TiO_*x*_ structure after post-annealing.

## Discussion

As earlier mentioned, in the nominal Pt/Ru/Co/TiO_*x*_ structure, post-annealing resulted in (1) A significant increase in *H*_c_ and VCC in thick *t*_Co_ regions, accompanied by an increase in the interfacial *m*_orb_. (2) Degradation of magnetisation, squareness, *H*_c_, and VCC in the thin *t*_Co_ regions. (3) Possible interdiffusion between Co and Pt, whereas Ru did not mix with Co or Pt. Based on these findings, we can discuss the changes in the Pt/Ru/Co/TiO_*x*_ structure that occurred during post-annealing.

First, regarding the enhancement of *H*_c_ upon annealing in the thick Co region, we discarded the fact that the PMA at the Pt/Ru/Co lower interface was improved owing to the mixture of Ru and Pt. Because the Ru layer remained undiffused, the interfacial *m*_orb_ and interface PMA are unlikely to increase significantly at the Pt/Ru/Co lower interface. It is more natural to assume that the *m*_orb_ and PMA at the Co/TiO_*x*_ upper interface are enhanced*.* The assumption is strongly supported by the significant enhancement in VCC by post-annealing, which mainly occurs at the Co/TiO_*x*_ upper interface. Previously, an increase in interfacial *m*_orb_ has been reported for Pt/Co/AlO_*x*_ trilayer but not in Pt/Co/Pt trilayer^30,31^. A post-annealing-induced PMA enhancement was also reported in Pt/Co/AlO_*x*_ trilayer but not in Pt/Co/Pt trilayer^29^. These results indicate that an interface PMA exist at Co/oxide interface and is enhanced by post-annealing, in Pt/Co/AlO_*x*_ trilayer. The enhancement may be interpreted as an improvement of interface PMA at the Co/oxide interface due to diffusion of Pt atoms into the vicinity of the Co/oxide interface. Since the Co/oxide interface exhibits PMA due to hybridization of Co 3*d* and O 2*p* orbitals despite the weak spin–orbit interaction (SOI)^7^, the incorporation of a heavy metal element with strong SOI, such as Pt, can enhance the interface PMA. Such enhancement of PMA at TM/oxide interface due to heavy mental elements incorporation has been reported both theoretically and experimentally^42–45^. In particular, enhancements of both PMA and VCMA at Co/oxide interface by Pt incorporation has been experimentally demonstrated^44,45^. In the structure of this study, we observed similar annealing temperature dependence of *H*_c_ (PMA) and VCC (VCMA) between Pt/Co/TiO_*x*_ and Pt/Ru/Co/TiO_*x*_ structures (Supplementary Information S5). The Ru layer inserted at the Pt/Co lower interface seems to have a limited effect on the post-annealing induced enhancement of PMA and VCC at the Co/TiO_*x*_ upper interface. The role of the Ru inserted layer is discussed in Supplementary Information S5. It is speculated that the post-annealing induced enhancement of *m*_orb_, PMA, and VCC occurs due to diffusion of Pt atoms into the vicinity of the Co/oxide interface for Pt/Ru/Co/TiO_*x*_ structure, as well. Furthermore, although not clearly visible in the EDX line profile (Fig. 2c), the diffusion of Pt atoms into Co layer is required to explain the annealing temperature dependence of VCC for Pt/Ru/Co/TiO_*x*_ structure. The post-annealing-induced change other than Pt atoms diffusion, such as diffusion of Co atoms into Pt layer and change in the oxidation state at the Co/oxide upper interface, are insufficient to explain the annealing temperature dependence of VCC. While a decrease of magnetisation cause increase of *H*_c_ and VCC, the at most 20% decrease (see Supplementary Information S2) of magnetisation due to Co diffusion alone does not cause the enhancement of VCC over several times. The diffusion of Co atoms into Pt layer can also affect the interface PMA at the Pt/Ru/Co lower interface. However, it will not significantly affect to the VCC at the Co/TiO_*x*_ upper interface. The post-annealing induced reduction of CoO at the Co/TiO_*x*_ upper interface (Fig. 3) can optimise the oxidation state and enhance PMA and VCMA (or VCC)^26–29,46^. However, such an enhancement of PMA and VCMA should occurs in the same way regardless the presence of Ru inserted layer. The differences in annealing temperature dependence of *H*_c_ and VCC between Pt/Co/TiO_*x*_ and Pt/Co/Ru/TiO_*x*_ structures (Fig. S5 in Supplementary Information S5) deny the dominant contribution of the optimisation of the oxidation state. From these considerations, we concluded the diffusion of Pt atoms into Co layer is required to explain the annealing temperature dependence of VCC in the Pt/Ru/Co/TiO_*x*_ structure.

Next, regarding the degradation of magnetisation, squareness, *H*_c_, and VCC in the thin Co region, the behaviour can be explained by considering the decrease in the Curie temperature (*T*_C_) of Co owing to post-annealing. The diffusion of nonmagnetic Pt atoms into Co layer decreases the *T*_C_ of Co^47^. The *T*_C_ reduction-induced degradation of the magnetic properties is significant in the thin Co region, where the *T*_C_ approximately equals the room temperature. This result indicates that excessive Pt diffusion degraded the magnetic properties, and an optimal annealing temperature exists for each Co thickness. Based on the above discussion, we speculated that a small amount of Pt-dispersed Co/oxide structure is key to obtain a large interface PMA and VCMA effect in fcc-Co-based stacks. Because post-annealing also improves the quality of the ferromagnetic layer/dielectric layer interface^48^, realising the aforementioned structure after post-annealing at appropriate temperatures is desirable. Similar to the present result, a large VCMA effect was obtained when a small amount of Ir was dispersed in the Fe layer in bcc-Fe-based stacks^49^. This similarity may indicate the effectiveness of the dispersion of 5*d* elements to obtain a large VCMA effect at TM ferromagnet/oxide dielectrics interfaces.

In fcc-Co(111) based stacks, a realisation of a giant tunnel magnetoresistance (TMR), in addition to a large VCMA effect, remains a challenge. Recently, interface-driven or band-folding-driven giant TMR were proposed based on first-principles calculations^50,51^, and its experimental demonstration is highly desired. We expect that the present study accelerate the material development of fcc-Co based stacks and lead to the realisation of the giant TMR.

## Conclusions

We systematically investigated the Co film thickness and annealing temperature dependence of voltage-controlled coercivity of Pt/Ru/Co/CoO/TiO_*x*_ structures and performed a multiprobe analyses, combining STEM-EDX, capacitance, and XAS/XMCD measurements. In the thick Co region (*t*_Co_ ~ 2.0 nm), we observed a significant increase in the voltage-controlled coercivity and enhancement in the interfacial *m*_orb_ by post-annealing and speculated that the diffusion of Pt atoms to the Co/oxide upper interface is a major contributor to the enhancement. On the other hand, in the thin Co region (1.5 nm ≤ *t*_Co_), we observed a degradation of the magnetic properties of Co upon post-annealing and speculated that the decrease in *T*_C_ of Co by Pt diffusion caused the degradation. Our results indicate that a small amount of Pt-dispersed Co/oxide structure is effective for achieving a large VCMA effect.

## Methods

Two types of samples were prepared using a combination of molecular beam epitaxy and sputtering techniques. The first is a sample with a wedge-shaped Co layer, which is formed using a linearly moving shutter during Co deposition. The nominal structure was a SiO_*x*_ sub./Ta (5 nm)/Ru (10 nm)/Ta (5 nm)/Pt (10 nm)/Ru (0.2 nm)/Co wedge (*t*_Co_ nm)/TiO_*x*_ (approximately 5 nm)/Pt (5 nm). The sample was microfabricated into an 8 × 10 μm^2^ pillar and used for MOKE measurements under bias-voltage and capacitance measurements. After microfabrication, the sample was annealed ex situ at up to 350 °C in a vacuum furnace. The deposition and microfabrication conditions were the same as previous studies^18^. The other was a sample with a Co layer of uniform thickness. The nominal structure was a SiO_*x*_ sub./Ta (5 nm)/Ru (10 nm)/Ta (5 nm)/Pt (10 nm)/Ru (0.2 nm)/Co (*t*_Co_ nm)/TiO_*x*_ (approximately 2 nm). These samples were used for VSM, STEM-EDX, and XAS/XMCD measurements and had a thinner cap layer for XAS/XMCD measurements.

The VCMA effects were investigated by measuring the perpendicular magnetisation curve with MOKE (wavelength = 408 nm) while applying a bias-voltage. The sign of the bias-voltage was defined with respect to the top electrode, indicating that a positive (negative) bias induced electron accumulation (depletion) at the Co/dielectric layer interface. Because *H*_c_ varies linearly with voltage^18^, the VCC in this study was estimated from the data obtained for a positive bias voltage (Δ*H*_c_/ΔV = *H*_c_@1 V − *H*_c_@0 V). The sign of VCC was consistent with that observed for surface-oxidised Pt/Co/oxide stacks^14–16^; *H*_c_ increased with positive voltage application or electron accumulation at the Co/dielectric layer interface. The dielectric layer thickness was not identified in this study. Thus, we divided the change in *H*_c_ by the voltage (V) rather than the electric field (V/nm). Because the results in Fig. 1 are for the same sample, the dielectric layer thickness is guaranteed to be the same, and a relative comparison of Δ*H*_c_/Δ*V* is possible. Semi-quantitative estimation of the VCMA coefficient for a similar structure has been reported previously^18^. The capacitances of the microfabricated samples were measured using an impedance analyser (Keysight, E4990A with a 42941A impedance probe). To obtain *C*/*S*, several devices with different sizes were measured and the parasitic capacitance components^38^ were subtracted. The magnetic properties (magnetisation and magnetic dead layer thickness) of the uniform films were measured using VSM.

The actual film structure was revealed using cross-sectional STEM and EDX analyses. *K*-(O, Ti, Co) or *L*-(Pt, Ru, Ta) edge element-specific fluorescence was employed in the EDX analysis. The EDX line profile was obtained by extracting the profile from the EDX element map (Supplementary Information S3). While the diameter of the beam spot was approximately 0.2 nm, the spectra were broadened owing to the spread of the electron beam within the sample (several nanometres).

XAS and XMCD were performed at BL-7A in the Photon Factory of the High Energy Accelerator Research Organization (KEK-PF). For XAS and XMCD measurements, the photon helicity was fixed, and a magnetic field of ± 1.2 T was applied parallel along the incident polarised soft X-ray beam to obtain signals defined as *μ*+ and *μ−* spectra. The total electron yield mode was adopted and all the measurements were performed at room temperature.

## Supplementary Information

Supplementary Information.

## Data Availability

The datasets generated during and/or analysed during the current study are available from the corresponding author on reasonable request.
