# Supplementary material for: Understanding voltage-controlled magnetic anisotropy effect at Co/oxide interface
Source: Sci Rep. 2023 Jun 30;13:10640. doi: 10.1038/s41598-023-37422-4 (PMC10313830; doi:10.1038/s41598-023-37422-4)
Supplement: Supplementary file 1 — Supplementary Information. [file 41598_2023_37422_MOESM1_ESM.pdf]

Supplementary Information

Understanding voltage-controlled magnetic anisotropy effect at Co/oxide interface

Tomohiro Nozaki[1*], Jun Okabayashi[2], Shingo Tamaru[1], Makoto Konoto[1],

Takayuki Nozaki[1], and Shinji Yuasa[1]

[1] National Institute of Advanced Industrial Science and Technology (AIST), Research Center for Emerging Computing Technologies (RCECT), Tsukuba, Ibaraki 305-8568, Japan

[2]Research Center for Spectrochemistry, The University of Tokyo, 113-0033, Tokyo, Japan

*email:nozaki.tomohiro@aist.go.jp

S1. t_{Co} dependence of magnetisation and Kerr rotation angle

Figure S1a shows the perpendicular magnetisation curve of as-deposited samples used for the X-ray absorption spectroscopy (XAS)/X-ray magnetic circular dichroism (XMCD) measurements, measured by vibrating sample magnetometer (VSM). Figure S1b shows the nominal Co thickness (t_{Co}) dependence of the areal magnetisation $M_{Co}t_{Co}$* extracted from Fig. S1a. Here M_{Co} and t_{Co}* represent magnetisation and assumed unoxidised thickness of Co, respectively. Figure S1c shows the t_{Co} dependence of the Kerr rotation angle θ_k of as-deposited sample used for magneto-optical Kerr effect (MOKE) measurement (same as Fig. 1e). Both the t_{Co} dependence of $M_{Co}t_{Co}$* and θ_k showed a liner dependence against t_{Co}. However, clear deviation from the linear trend was found in the thin Co regions. The deviation suggests the enhanced oxidation resistance of Co in the thin Co regions, possibly due to the mixing with Ru and/or Pt. Therefore, the deviated region was excluded from the estimation of the magnetic dead layer thickness t_{dead}. We estimated the t_{dead} to be 1.0 nm from the linear fitting of t_{Co} = 1.5 nm and 2.0 nm data of Fig. S1b. The slope, 1.2×10^6 A/m is consistent with our previous reports[S1], ensuring the certainty of the fitting results. θ_k showed a similar t_{Co} dependence with $M_{Co}t_{Co}$*, but the intercept differs. Thus, estimating the t_{dead} from the MOKE measurements results in under-estimation, possibly due to the difference in Kerr rotation angle between Co and Ru/Pt. When compared within the same sample, roughly, the smaller the θ_k, the smaller the magnetisation. However, it should be noted that the absolute value of the θ_k is not completely proportional to the magnetisation.

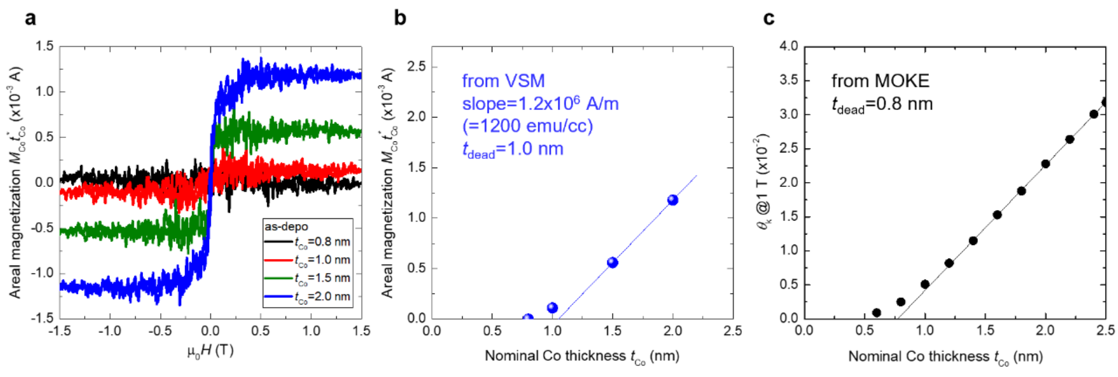

Figure S1. (**a**) Perpendicular magnetisation curve of as-deposited samples used for XAS/XMCD measurements, measured by VSM. (**b**) and (**c**) t_{Co} dependence of the (**b**) $M_{Co}t_{Co}$* and (**c**) θ_k of as-deposited sample.

S2. Anneal temperature dependence of magnetisation and Kerr rotation angle

Here we compared the post-anneal temperature dependence of the magnetisation and the θ_k of the nominal SiO_x sub./Ta (5 nm)/Ru (10 nm)/Ta (5 nm)/Pt (10 nm)/Ir (0.2 nm)/Co (2 nm)/TiO_x (approximately 3 nm)/Pt (5 nm) structure (Fig. S2a). The deposition conditions are the same as previous studies[S1]. Figure S2b compare the anneal temperature dependence of $M_{Co}t_{Co}$* (black circles) and θ_k (blue circles) of the sample. Figure S2c,d shows the corresponding perpendicular magnetisation curves measured by VSM and MOKE for various annealing temperature. In this sample, annealing at 200 °C results in a decrease in magnetisation of approximately 20 %. Further annealing up to 400 °C cause little change in magnetisation (Fig. S2b,c). On the other hand, the θ_k showed a different annealing temperature dependence. After decrease at 200-°C-annealing, a marked increase in the θ_k above 350-°C-annealing was observed (Fig. S2b,d). This increase may be the result of the Pt diffusion. Thus, the θ_k roughly correspond to magnetisation. However, it should be noted that the annealing temperature dependence of magnetisation cannot be discussed quantitatively from the θ_k.

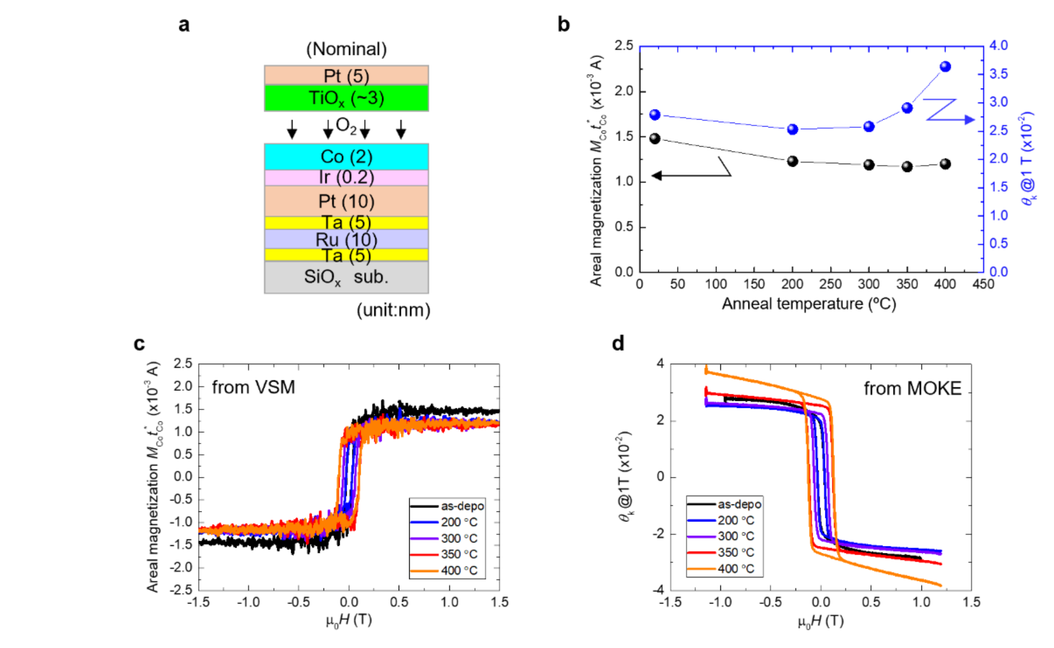

Figure S2. (**a**) Schematics of nominal structure of the sample for the magnetisation curve measurements. (**b**) Anneal temperature dependence of $M_{Co}t_{Co}$* and θ_k. (**c**) and (**d**) Perpendicular magnetisation curves measured by (**c**) VSM and (**d**) MOKE for various annealing temperature.

S3 EDX element map

Figure S3b,c show the energy dispersive X-ray spectroscopy (EDX) element map of as-deposited and 350-ºC-annealed samples of the nominal SiO_x sub./Ta (5 nm)/Ru (10 nm)/Ta (5 nm)/Pt (10 nm)/Ru (0.2 nm)/Co (2 nm)/TiO_x (approximately 2 nm) structure. The schematics of the structure was shown in Fig. S3a. The element map is almost identical before and after post-annealing, and no significant change can be identified. See main text for a more detailed discussion based on the line profiles.

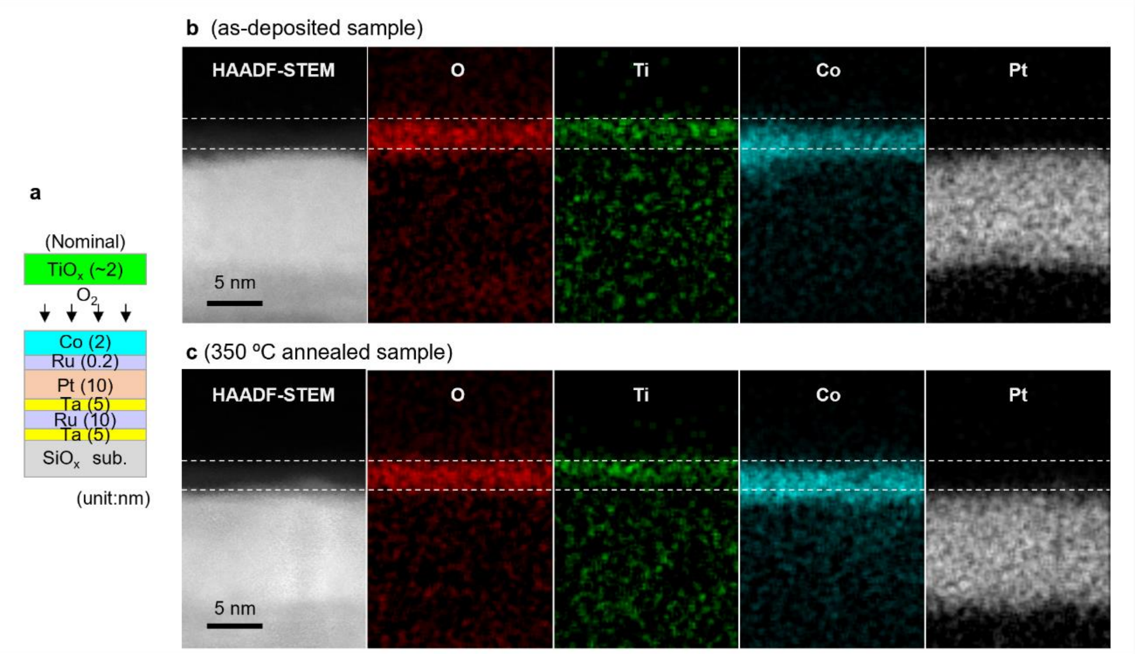

Figure S3. (**a**) Schematics of nominal structure of the sample for the STEM-EDX analysis. (**b**) and (**c**) EDX element map of (**b**) as-deposited and (**c**) 350-ºC-annealed samples.

S4 Comparison of XAS spectra

Figure S4 compare the normalised Co *L*-edge XAS spectra of as-deposited and 350-ºC-annealed samples of the nominal SiO_x sub./Ta (5 nm)/Ru (10 nm)/Ta (5 nm)/Pt (10 nm)/Ru (0.2 nm)/Co (2 nm)/TiO_x (approximately 2 nm) structure. The XPS spectrum of SiO_x sub./Ta (5 nm)/Ru (10 nm)/Ta (5 nm)/Pt (10 nm)/Co (1.3 nm)/MgO (2.0 nm) structure is also shown, as a reference spectrum of sample without CoO. The CoO component at 778.0 eV decreased by the post-annealing. On the other hand, the spectrum of the 350-ºC-annealed sample is clearly broadened compared to the sample without CoO (Pt/Co/MgO), indicating that the CoO component remains after the post-annealing.

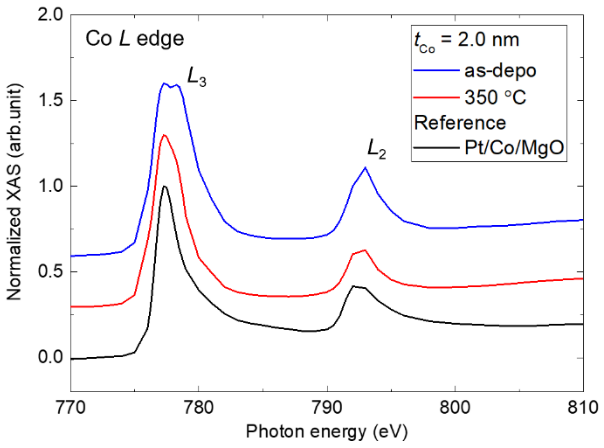

Figure S4. Normalised Co *L*-edge XAS spectra $((\mu- + \mu+)/2)$ of as-deposited and 350-ºC-annealed samples for t_{Co} = 2.0 nm. The XAS spectra of Pt/Co/MgO structure was also shown as a reference spectrum of sample without CoO.

S5 Annealing temperature dependence of $\Delta H_c/\Delta V$ and H_c for samples without and with Ru layer insertion

Figure S5b,c compare the annealing temperature dependence of voltage-controlled H_c (VCC = $\Delta H_c/\Delta V$) and H_c for Pt/Co/TiO_x (without insertion) and Pt/Ru/Co/TiO_x (with Ru layer insertion) structures[S1]. The schematics of the nominal structure (SiO_x sub./Ta (5 nm)/Ru (10 nm)/Ta (5 nm)/Pt (10 nm)/Ru (0 or 0.2 nm)/Co (1.7 nm)/TiO_x (approximately 5 nm)/Pt (5 nm) structures) was shown in Fig. S5a. In Fig. S5b, the overall trend of VCC were similar for both samples; with increasing annealing temperature, first VCC increase, and have a maximum at a certain temperature (indicated by black and blue arrows), and then degrade. The change in VCC can be explained from post-annealing induced diffusion of Pt atoms into Co layer. As discussed in the main text, Pt atoms diffused into the vicinity of Co/oxide interface cause the enhancement of PMA (H_c) and VCMA (VCC). However, excessive diffusion of Pt atoms into Co layer cause the decrease of Curie temperature (T_c) of Co[S2], leading to the degrade of H_c and VCC. Thus H_c and VCC maximised at a certain annealing temperature and then degrade. The similar overall trend of annealing temperature dependence of VCC suggests the occurrence of Pt atoms diffusion into Co layer for both Pt/Co/TiO_x and Pt/Ru/Co/TiO_x structures, regardless the presence of Ru inserted layer. On the other hand, we found two distinct differences between these structures. Compared with Pt/Ru/Co/TiO_x structure, we observed a larger VCC from a lower annealing temperature for Pt/Co/TiO_x structure, indicating a larger Pt atoms mixing and Pt atoms diffusion into Co layer. We observed a degradation of VCC from a lower annealing temperature for Pt/Co/TiO_x structure, indicating a lower T_c due to a larger Pt atoms diffusion into Co layer. Similar trends were also observed for the annealing temperature dependence of H_c (Fig. S5c). From these results, we concluded that the Pt diffusion into Co layer occurs even in the Pt/Ru/Co/TiO_x structures (with Ru layer insertion). However, the degree of mixing and interdiffusion is small in the Pt/Ru/Co/TiO_x structure compared with the Pt/Co/TiO_x structure. We interpreted the role of the 0.2 nm (one monolayer) Ru layer inserted at the Pt/Co interface as weaken, but does not completely prevent interdiffusion of Pt and Co.

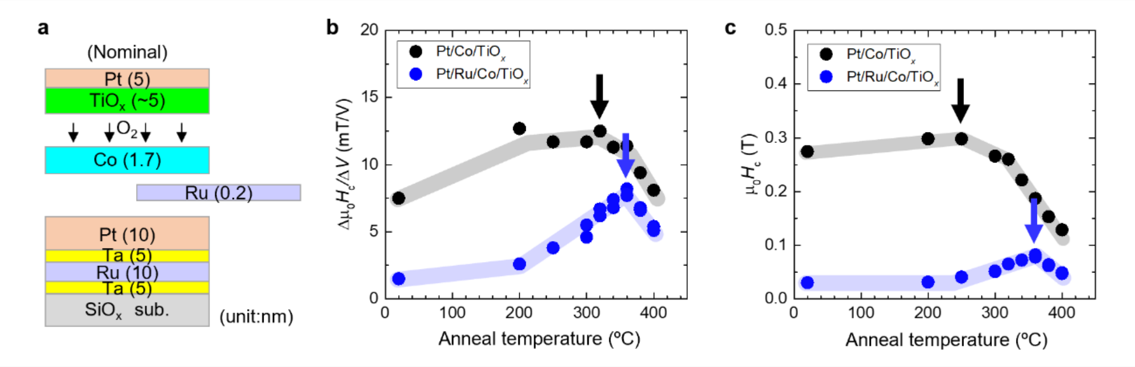

Figure S5. (**a**) Schematics of nominal structure of the samples. (**b**) and (**c**) Annealing temperature dependence of (**b**) $\Delta H_c/\Delta V$ and (**c**) H_c for Pt/Co/TiO_x (without insertion) and Pt/Ru/Co/TiO_x (with Ru layer insertion) structures[S1].

References

S1. Nozaki, T., Tamaru, S., Konoto, M., Nozaki, T., Kubota, H., Fukushima, A., and Yuasa, S., Large voltage-induced coercivity change in Pt/Co/CoO/amorphous TiO_x structure and heavy metal insertion effect. *Sci. Rep.* **11**, 21448 (2021).

S2. Constant, F. W. The Magnetic Properties of Certain Pt-Co and Pd-Co Alloys. *Phys. Rev.* **36**, 1654 (1930).
